# Supplementary material for: Functional and molecular characterization of a non-human primate model of autism spectrum disorder shows similarity with the human disease
Source: Nat Commun. 2021 Sep 15;12:5388. doi: 10.1038/s41467-021-25487-6 (PMC8443557; doi:10.1038/s41467-021-25487-6)
Supplement: Supplementary file 1 — Supplementary Information [file 41467_2021_25487_MOESM1_ESM.pdf]

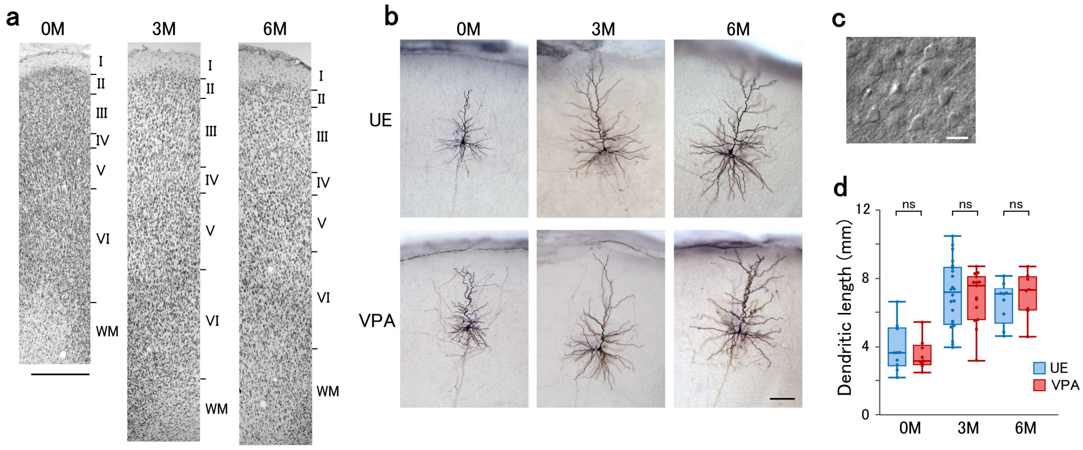

Supplementary Figure 1. Cytoarchitecture and structure of layer 3 pyramidal neurons in the dorsomedial prefrontal cortex.

(a) Nissl staining of area 8b in UE animals at 0M, 3M, and 6M. Scale bar, 200 μm. These images are representative of 5 animals for each age. I-VI, cortical layers 1-6; WM, white matter.

(b) Photomicrographs of biocytin-stained layer 3 pyramidal neurons from UE and VPA animals at 0M, 3M, and 6M. Scale bar, 100 μm. These images are representative of 10 cells (0M UE), 9 cells (0M VPA), 22 cells (3M UE), 15 cells (3M VPA), 9 cells (6M UE), and 8 cells (6M VPA).

(c) IR-DIC image of layer 3 of the slice from a UE animal at 3M (representative of 21 slices). Scale bar, 20 μm.

(d) Total dendritic length of layer 3 pyramidal neurons from UE (blue) and VPA (red) animals. The box plots represent the median, quartiles, and data range. $n = 10$ cells in 4 animals (0M UE), $n = 9$ cells in 2 animals (0M VPA), $n = 22$ cells in 4 animals (3M UE), $n = 15$ cells in 3 animals (3M VPA), $n = 9$ cells in 3 animals (6M UE), and $n = 8$ cells in 2 animals (6M VPA). Two-sided t-test with Holm-Sidak correction between UE and VPA, $p = 0.75$ (0M), $p = 0.79$ (3M), and $p = 0.86$ (6M). ns: not significant.

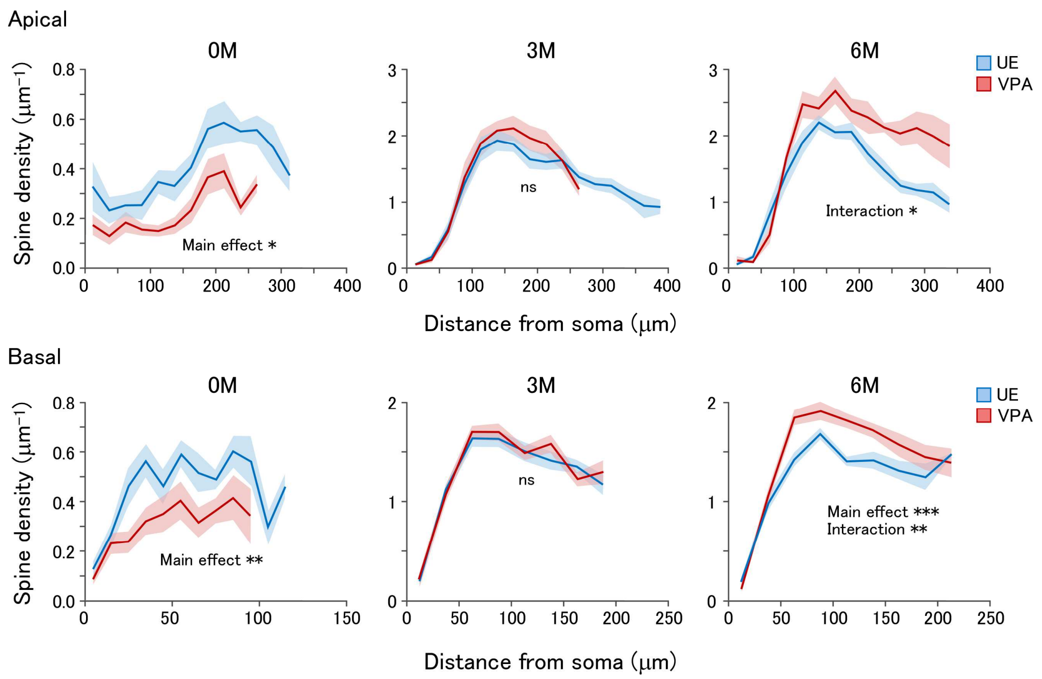

Supplementary Figure 2. Spine density along the entire length of apical and basal dendrites.

The average spine density is shown by the lines, and the SEM is shown by the shaded area. For apical dendrites at 0M, $n = 9$ dendrites in 4 animals (UE) and $n = 8$ dendrites in 2 animals (VPA); two-way repeated-measures ANOVA, main effect of treatment, $F(1,13) = 5.52$, $p = 0.035$; interaction of distance × treatment, $F(5,65) = 0.80$, $p = 0.55$. At 3M, $n = 11$ dendrites in 5 animals (UE) and $n = 9$ dendrites in 3 animals (VPA); main effect, $F(1,17) = 1.37$, $p = 0.26$; interaction, $F(5,85) = 0.41$, $p = 0.84$. At 6M, $n = 15$ dendrites in 4 animals (UE) and $n = 8$ dendrites in 3 animals (VPA); main effect, $F(1,21) = 2.51$, $p = 0.13$; interaction, $F(5,105) = 2.83$, $p = 0.019$. For basal dendrites at 0M, $n =14$ dendrites in 9 cells, 4 animals (UE) and $n = 18$ dendrites in 12 cells, 3 animals (VPA); main effect, $F(1,21) = 8.84$, $p = 0.0072$; interaction, $F(4,84) = 0.18$, $p = 0.95$. At 3M, $n = 17$ dendrites in 10 cells, 4 animals (UE) and $n = 14$ dendrites in 6 cells, 3 animals (VPA); main effect, $F(1,28) = 0.050$, $p = 0.83$; interaction, $F(3,84) = 0.60$, $p = 0.61$. At 6M, $n = 20$ dendrites in 14 cells, 4 animals (UE) and $n = 22$ dendrites in 7 cells, 3 animals (VPA); main effect, $F(1,40) = 14.0$, $p = 0.00056$; interaction, $F(3,120) = 4.12$, $p = 0.0080$. P-values for the main effect and interaction are shown. $^{***}p < 0.001$; $^{**}p < 0.01$; $^{*}p < 0.05$; *ns* indicates that neither the main effect nor the interaction is significant.

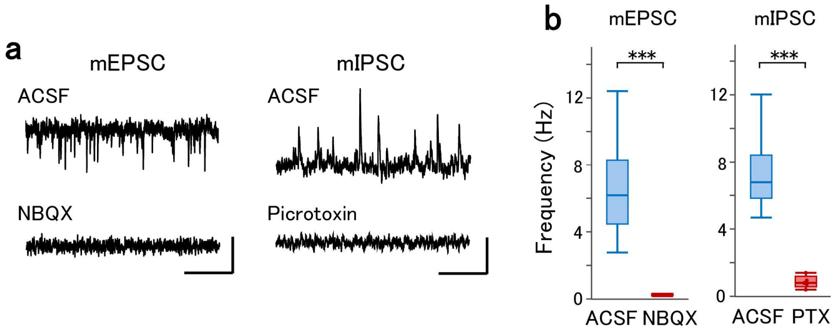

Supplementary Figure 3. Effects of AMPA and $GABA_A$ receptor blockers on miniature synaptic currents in UE animals at 3M.

(a) Representative traces of mEPSCs in normal ACSF and in the presence of NBQX (left), as well as mIPSCs in normal ACSF and in the presence of picrotoxin (right).

(b) NBQX effects on the frequency of mEPSCs (left; two-sided *t*-test, $p = 6.9 \times 10^{-11}$) and picrotoxin effects on the frequency of mIPSCs (right; two-sided *t*-test, $p = 1.6 \times 10^{-22}$). The box plots represent the median, quartiles, and data range. $n = 28$ cells in 6 animals (mEPSCs, ACSF), $n = 5$ cells in 2 animals (mEPSCs, NBQX), $n = 37$ cells in 7 animals (mIPSCs, ACSF), and $n = 5$ cells in 2 animals (mIPSCs, picrotoxin). The data in ACSF are the same as in Figure 1. $^{***}p < 0.001$.

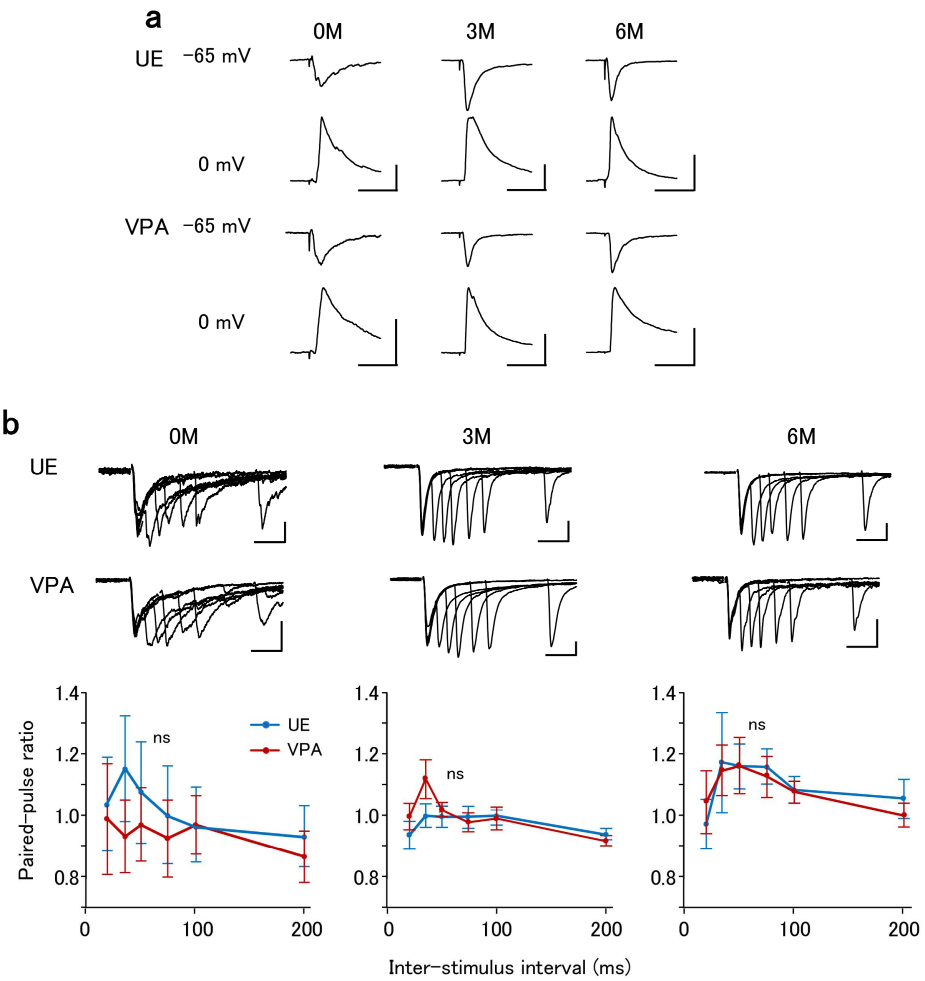

Supplementary Figure 4. EPSCs and IPSCs evoked by stimulation of layers 4-5, as well as the effects of VPA exposure on the paired-pulse ratio of evoked EPSCs.

(a) Representative traces of stimulus-evoked EPSCs and IPSCs in UE and VPA animals at 0M, 3M, and 6M. The stimulus intensity was 300 μA. Vertical scale bars: 200 pA for EPSCs and 400 pA for IPSCs at 0M; 500 pA for EPSCs and 1000 pA for IPSCs at 3M and 6M. Horizontal scale bars, 50 ms.

(b) EPSCs evoked by paired-pulse stimulation (inter-stimulus interval 20-200 ms) in UE (top) and VPA (bottom) animals at 0M, 3M, and 6M. Vertical scale bars, 50 pA for 0M, and 200 pA for 3 and 6M. Horizontal scale bars, 50 ms. The paired-pulse ratios in UE (blue) and VPA (red) animals are plotted as a function of the inter-stimulus interval. The error bars represent the SEM. At 0M, $n = 17$ cells in 6 animals (UE) and $n = 10$ cells in 2 animals (VPA); two-way repeated-measures ANOVA, main effect of treatment, $F(1,25) = 0.17$, $p = 0.69$; interaction of intensity × treatment, $F(4,100) = 0.77$, $p = 0.55$. At 3M, $n = 23$ cells in 5 animals (UE) and $n = 14$ cells in 2 animals (VPA); main effect, $F(1,35) = 0.69$, $p = 0.41$; interaction, $F(4,140) = 1.96$, $p = 0.10$. At 6M, $n = 12$ cells in 2 animals (UE) and $n = 11$ cells in 2 animals (VPA); main effect,

$F(1,21) = 0.0002$, $p = 0.99$, interaction, $F(4,84) = 0.30$, $p = 0.88$. *ns* indicates that neither the main effect nor the interaction is significant.

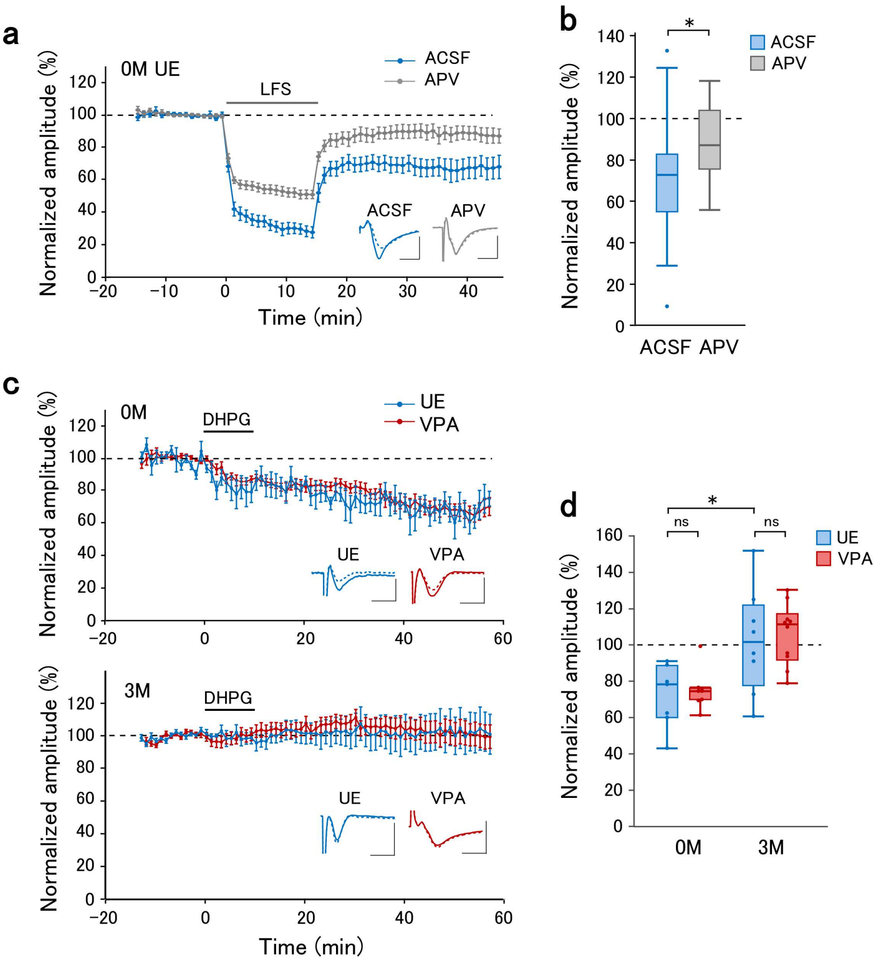

Supplementary Figure 5. Dependency of LFS-induced LTD on NMDA receptors and the effects of VPA exposure on mGluR-dependent LTD.

(a) The time course of the normalized field EPSP amplitudes with low-frequency stimulation (LFS) in UE animals at 0M in normal ACSF (blue) and in the presence of APV (gray). The error bars represent the SEM. Insets are representative average traces before (−10 to 0 min, solid lines) and after (30 to 40 min, dotted lines) LFS. $n = 26$ pathways in 12 animals (ACSF) and $n = 17$ pathways in 3 animals (APV).

(b) Normalized EPSP amplitudes in normal ACSF and in the presence of APV after LTD induction (30 to 40 min). The box plots represent the median, quartiles, and data range. Two-sided t-test, $p = 0.030$. The data in normal ACSF are the same as in Figure 2. $*p < 0.05$.

(c) The time course of the normalized EPSP amplitudes with DHPG stimulation in UE (blue) and VPA (red) animals at 0M and 3M. LTD was induced by perfusion of DHPG (100 μM) for 10 min. The error bars represent the SEM. Insets are representative average traces before (−10 to 0 min, solid lines) and after (30 to 40 min, dotted lines) DHPG application. $n = 7$ pathways in 2 animals (0M UE), $n = 8$ pathways in 1 animal (0M VPA), $n = 8$ pathways in 3 animals (3M UE), and $n = 10$ pathways in 2 animals

(3M VPA).

(d) Normalized EPSP amplitudes at 30-40 min after the onset of DHPG perfusion. The box plots represent the median, quartiles, and data range. Two-sided *t*-test with Holm-Sidak correction between UE and VPA, $p = 0.89$ (0M) and $p = 0.74$ (3M). Two-sided *t*-test between 0M and 3M in UE animals, $p = 0.029$. *$p < 0.05$; ns: not significant.

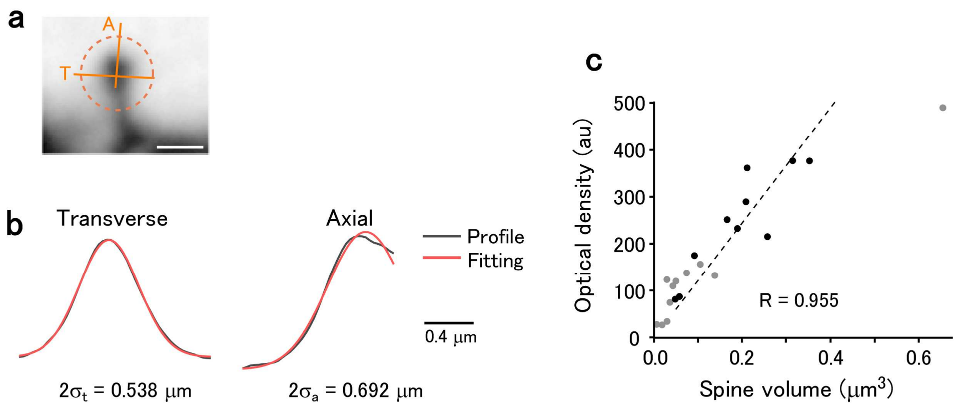

Supplementary Figure 6. Measurement of spine volumes in biocytin-filled neurons.
(a) Representative image of a dendritic spine. Optical density profiles along the transverse (T) and axial (A) lines are shown in **(b)**. Scale bar, 1 μm.
(b) Optical density profiles along the lines shown in **(a)** (black) and their Gaussian fitting (red).
(c) Relationship between the calculated spine volume and the optical density in 21 spines on a dendrite. The dotted line represents the linear regression between the optical density and volume for spines with a diameter > 0.4 μm and volume < 0.4 μm^3 (black dots).

a

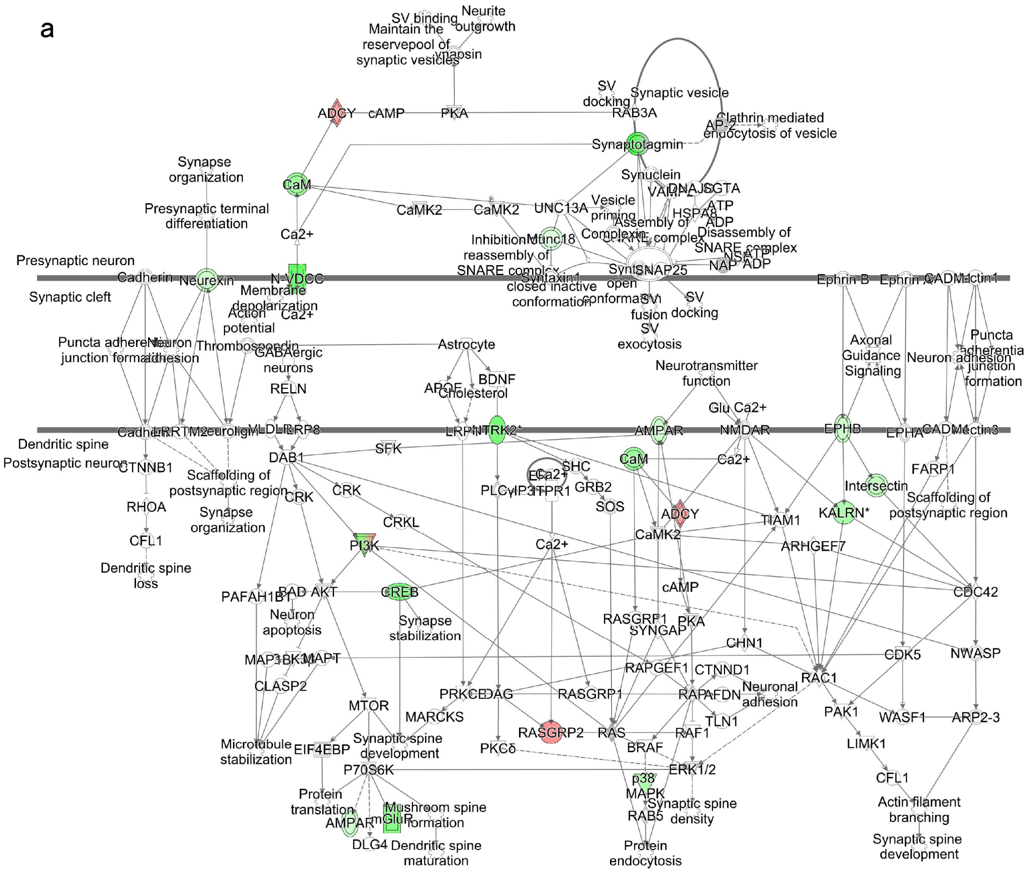
Neurite outgrowth
SV binding
Maintain the reservepool of synaptic vesicles
ADCY
cAMP
PKA
SV docking
Synaptic vesicle
RAB3A
Clathrin mediated endocytosis of vesicle
Synaptotagmin
Synapse organization
CaM
Presynaptic terminal differentiation
CaMK2
UNC13A
Vesicle priming
Synuclein
ATP
ADP
Disassembly of SNARE complex
Ca2+
Inhibition of reassembly of SNARE complex
Presynaptic neuron
Synaptic cleft
Cadherin
Neurexin
Membrane depolarization
Action potential
SNAP25
SV fusion
SV docking
SV exocytosis
Ephrin B
Puncta adherens junction formation
Thrombospondin
GABAergic neurons
Astrocyte
BDNF
RELN
Cholesterol
Neurotransmitter function
Axonal Guidance Signaling
Neuron adhesion
Puncta adherentia junction formation
Glu Ca2+
AMPAR
NMDAR
EPHB
Dendritic spine
Postsynaptic neuron
CTNNB1
DAB1
SFK
SHC
GRB2
SOS
Scaffolding of postsynaptic region
CRK
RHOA
Synapse organization
CRKL
PLCγ
ADCY
TIAM1
Intersectin
KALRN*
FARP1
Scaffolding of postsynaptic region
CFL1
Dendritic spine loss
PI3K
Ca2+
ARHGEF7
PAFAH1B1
AKT
CREB
cAMP
CDC42
Neuron apoptosis
Synapse stabilization
RASGRF1
PKA
SYNGAP
CHN1
CDK5
NWASP
MAPT
CLASP2
RAPGEF1
CTNND1
RAC1
MTOR
PRKCE
DAG
RASGRP1
Neuronal adhesion
PAK1
WASF1
ARP2-3
MARCKS
RASGRP2
RAS
RAF1
TLN1
Microtubule stabilization
EIF4EBP
Synaptic spine development
PKCδ
BRAF
LIMK1
P70S6K
ERK1/2
p38 MAPK
CFL1
Protein translation
Mushroom spine formation
Synaptic spine density
RAB5
Actin filament branching
AMPAR
DLG4
Dendritic spine maturation
Protein endocytosis
Synaptic spine development

b

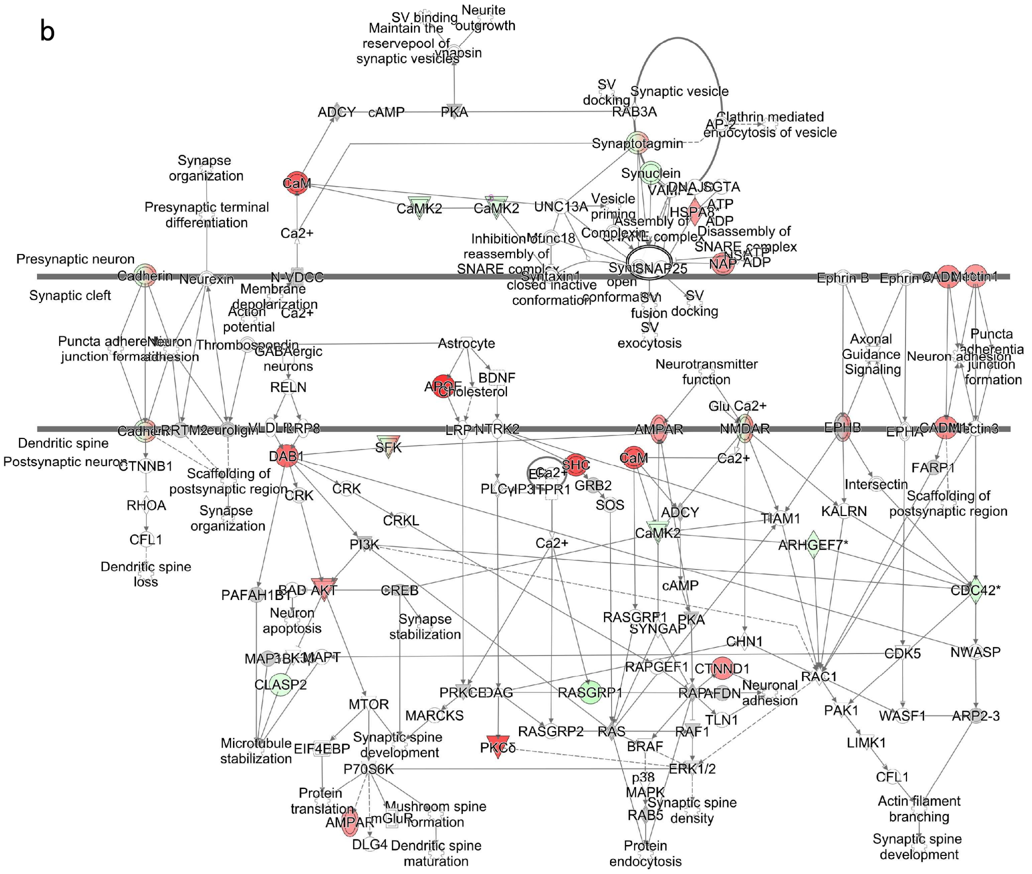
Neurite outgrowth
SV binding
Maintain the reservepool of synaptic vesicles
ADCY
cAMP
PKA
SV docking
Synaptic vesicle
RAB3A
Clathrin mediated endocytosis of vesicle
Synaptotagmin
Synapse organization
CaM
Presynaptic terminal differentiation
CaMK2
UNC13A
Vesicle priming
Synuclein
ATP
ADP
Disassembly of SNARE complex
Ca2+
Inhibition of reassembly of SNARE complex
Presynaptic neuron
Synaptic cleft
Cadherin
Neurexin
Membrane depolarization
Action potential
SNAP25
SV fusion
SV docking
SV exocytosis
Ephrin B
Puncta adherens junction formation
Thrombospondin
GABAergic neurons
Astrocyte
BDNF
RELN
Cholesterol
Neurotransmitter function
Axonal Guidance Signaling
Neuron adhesion
Puncta adherentia junction formation
Glu Ca2+
AMPAR
NMDAR
EPHB
Dendritic spine
Postsynaptic neuron
CTNNB1
DAB1
SFK
SHC
GRB2
SOS
Scaffolding of postsynaptic region
CRK
RHOA
Synapse organization
CRKL
PLCγ
ADCY
TIAM1
Intersectin
KALRN
FARP1
Scaffolding of postsynaptic region
CFL1
Dendritic spine loss
PI3K
Ca2+
ARHGEF7*
PAFAH1B1
AKT
CREB
cAMP
CDC42*
Neuron apoptosis
Synapse stabilization
RASGRF1
PKA
SYNGAP
CHN1
CDK5
NWASP
MAPT
CLASP2
RAPGEF1
CTNND1
RAC1
MTOR
PRKCE
DAG
RASGRP1
Neuronal adhesion
PAK1
WASF1
ARP2-3
MARCKS
RASGRP2
RAS
RAF1
TLN1
Microtubule stabilization
EIF4EBP
Synaptic spine development
PKCδ
BRAF
LIMK1
P70S6K
ERK1/2
p38 MAPK
CFL1
Protein translation
Mushroom spine formation
Synaptic spine density
RAB5
Actin filament branching
AMPAR
DLG4
Dendritic spine maturation
Protein endocytosis
Synaptic spine development

Supplementary Figure 7. Distinct gene expression modulations in the synaptogenesis signaling pathway at 0M and 3M by the IPA software.

(a) Modulated gene expression at 0M. Molecules filled in red are upregulated, and those filled in green are downregulated.

(b) Modulated gene expression at 3M.

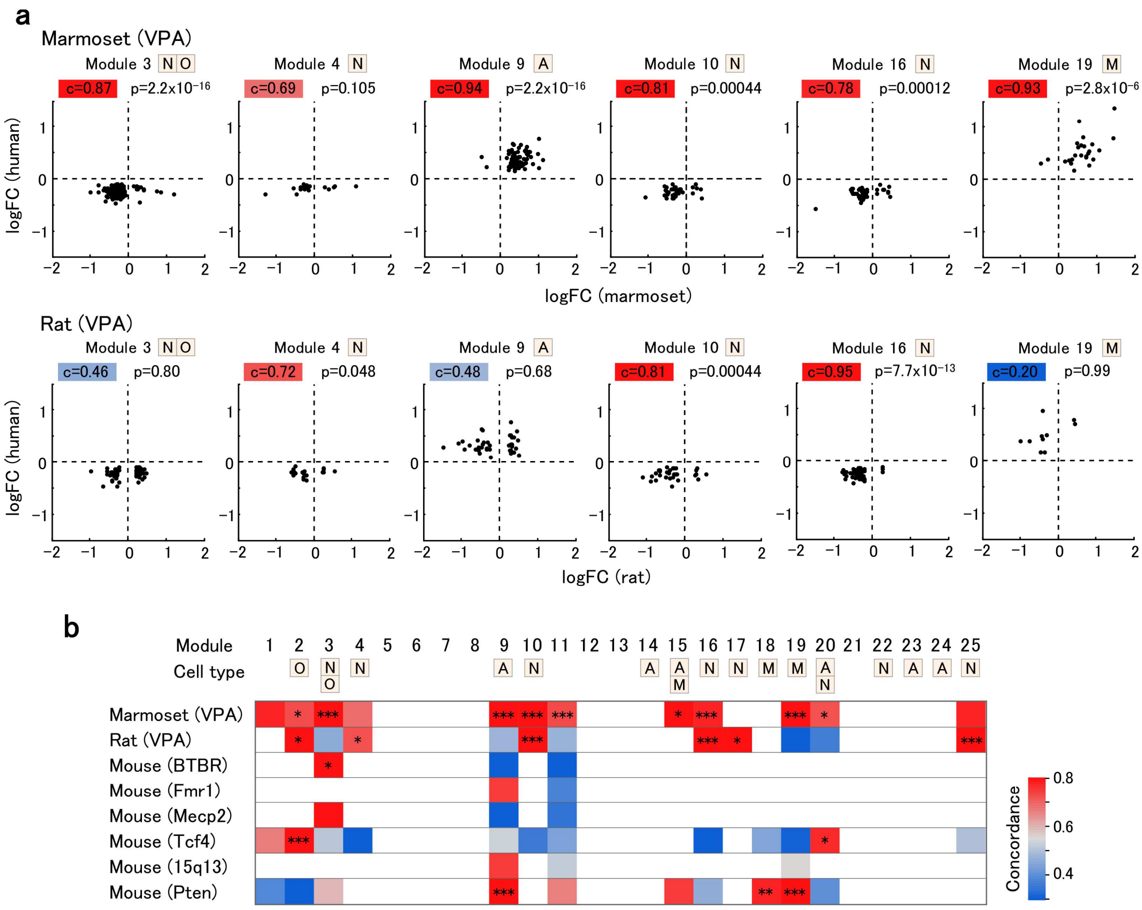

Supplementary Figure 8. Correlation between the logFC values in marmosets at 3M or rodent models and human ASD analyzed for each coexpression module.

(a) Gene expression modulations in the marmoset VPA model at 3M (top) and rat VPA model[1] (bottom) plotted against human ASD[2]. Genes with p_{adj} < 0.05 for rats and p_{adj} < 0.1 for marmosets and humans are plotted. Modules with at least 10 common genes for both models are shown. A, M, N, and O denote modules related to astrocytes, microglia, neurons, and oligodendrocytes, respectively. Concordance (number of genes showing concordant changes between the animal model and human ASD divided by the total number of genes, colored as in **(b)**) and p-values from the one-sided binomial test are shown.

(b) Concordance of gene expression modulations for each human module. For modules with at least 8 common genes between the animal model and human ASD, concordance is shown by the color. Asterisks represent the p-values with the one-sided binomial test: *p<0.05, **p<0.01, ***p<0.001. Exact p-values are in the Source Data. The rat VPA model is at 35 days of age[1]. The mouse models are from the BTBR strain at 4 months[3], *Fmr1* knockout mice at 8-14 weeks[4], *Mecp2* heterozygous mice at 5 weeks[5], *Tcf4* mutant mice (combination of multiple models) at 60-80 days[6], 15q13 homozygous mutant mice at 10-22 weeks[7], and *Pten*^{m3m4} mice at 6 weeks[8]. For the rat VPA model and BTBR mice,

the genes with $p_{adj} < 0.05$ were selected; for other models and human ASD, $p_{adj} < 0.1$ were selected. Mouse models of maternal immune activation[9] and *Shank3* knockout[10] were not included in the list, because of the small number of commonly affected genes.

References

1. Zhang, R. *et al.* Transcriptional and splicing dysregulation in the prefrontal cortex in valproic acid rat model of autism. *Reprod. Toxicol.* **77**, 53–61 (2018).
2. Parikshak, N. N. *et al.* Genome-wide changes in lncRNA, splicing, and regional gene expression patterns in autism. *Nature* **540**, 423–427 (2016).
3. Mizuno, S. *et al.* Comprehensive profiling of gene expression in the cerebral cortex and striatum of BTBRTF/ArtRbrc mice compared to C57BL/6J mice. *Front. Cell. Neurosci.* **14**, 1–24 (2020).
4. Rogers, T. D. *et al.* Effects of a social stimulus on gene expression in a mouse model of fragile X syndrome. *Mol. Autism* **8**, 30 (2017).
5. Zhao, D. *et al.* Transcriptome analysis of microglia in a mouse model of Rett syndrome: differential expression of genes associated with microglia/macrophage activation and cellular stress. *Mol. Autism* **8**, 17 (2017).
6. Phan, B. N. *et al.* A myelin-related transcriptomic profile is shared by Pitt–Hopkins syndrome models and human autism spectrum disorder. *Nat. Neurosci.* **23**, 375–385 (2020).
7. Gordon, A. *et al.* Transcriptomic networks implicate neuronal energetic abnormalities in three mouse models harboring autism and schizophrenia-associated mutations. *Mol. Psychiatry* **26**, 1520–1534 (2021).
8. Tilot, A. K. *et al.* Neural transcriptome of constitutional Pten dysfunction in mice and its relevance to human idiopathic autism spectrum disorder. *Mol. Psychiatry* **21**, 118–125 (2016).
9. Smith, S. E. P., Li, J., Garbett, K., Mirnics, K. & Patterson, P. H. Maternal immune activation alters fetal brain development through interleukin-6. *J. Neurosci.* **27**, 10695–10702 (2007).
10. Qin, L. *et al.* Social deficits in Shank3-deficient mouse models of autism are rescued by histone deacetylase (HDAC) inhibition. *Nat. Neurosci.* **21**, 564–575 (2018).
