# Supplementary material for: Functional and molecular characterization of a non-human primate model of autism spectrum disorder shows similarity with the human disease
Source: Nat Commun. 2021 Sep 15;12:5388. doi: 10.1038/s41467-021-25487-6 (PMC8443557; doi:10.1038/s41467-021-25487-6)
Supplement: Supplementary file 3 — Description of Additional Supplementary Files [file 41467_2021_25487_MOESM3_ESM.docx]

Description of Additional Supplementary Files

Title: Supplementary Data 1.

Description: Summary of the number of animals and samples used in each experiment.

Title: Supplementary Data 2.

Description: List of affected genes categorized into three clusters. The logFC and padj (Benjamini-Hochberg-adjusted values of two-sided t-test) in VPA-exposed marmosets at each time point, as well as the module, logFC, and padj in human ASD 48, are shown.

Title: Supplementary Data 3.

Description: Critical period-related genes. Critical period-related genes listed in two studies 46, 47 and expressed in the marmoset brain. Cluster numbers are shown for marmoset DEGs.

Title: Supplementary Data 4.

Description: Pathway analysis of the gene clusters. The p values and the ratio are as provided by the IPA software.
