# Supplementary material for: Functional and molecular characterization of a non-human primate model of autism spectrum disorder shows similarity with the human disease
Source: Nat Commun. 2021 Sep 15;12:5388. doi: 10.1038/s41467-021-25487-6 (PMC8443557; doi:10.1038/s41467-021-25487-6)
Supplement: Supplementary file 8 — Reporting summary [file 41467_2021_25487_MOESM8_ESM.pdf]

nature research

Corresponding author(s): Noritaka Ichinohe
Satoshi Watanabe

Last updated by author(s): Jul 30, 2021

Reporting Summary

Nature Research wishes to improve the reproducibility of the work that we publish. This form provides structure for consistency and transparency in reporting. For further information on Nature Research policies, see our Editorial Policies and the Editorial Policy Checklist.

Statistics

For all statistical analyses, confirm that the following items are present in the figure legend, table legend, main text, or Methods section.

n/a	Confirmed	
☐	☒	The exact sample size (n) for each experimental group/condition, given as a discrete number and unit of measurement
☐	☒	A statement on whether measurements were taken from distinct samples or whether the same sample was measured repeatedly
☐	☒	The statistical test(s) used AND whether they are one- or two-sided<br>*Only common tests should be described solely by name; describe more complex techniques in the Methods section.*
☒	☐	A description of all covariates tested
☐	☒	A description of any assumptions or corrections, such as tests of normality and adjustment for multiple comparisons
☐	☒	A full description of the statistical parameters including central tendency (e.g. means) or other basic estimates (e.g. regression coefficient) AND variation (e.g. standard deviation) or associated estimates of uncertainty (e.g. confidence intervals)
☐	☒	For null hypothesis testing, the test statistic (e.g. F, t, r) with confidence intervals, effect sizes, degrees of freedom and P value noted<br>*Give P values as exact values whenever suitable.*
☒	☐	For Bayesian analysis, information on the choice of priors and Markov chain Monte Carlo settings
☒	☐	For hierarchical and complex designs, identification of the appropriate level for tests and full reporting of outcomes
☐	☒	Estimates of effect sizes (e.g. Cohen's d, Pearson's r), indicating how they were calculated

Our web collection on statistics for biologists contains articles on many of the points above.

Software and code

Policy information about availability of computer code

Data collection	Igor Pro (WaveMetrics, v6.0); Neurolucida (MBF Bioscience, v10 and v11).
Data analysis	Mini Analysis (Synaptosoft, v6.0.3), MATLAB (MathWorks, v7.9 and v9.10) with custom codes; Neurolucida (MBF Bioscience, v10 and v11), Praat (www.praat.org, v6.1); MAS5 and GCRMA (Bioconductor) on R (v3.6); IPA (Qiagen, Summer Release 2020); EZR (https://www.jichi.ac.jp/saitama-sct/SaitamaHP.files/statmed.html). Custom codes are available at https://github.com/ncnp-bisai/matlab.

For manuscripts utilizing custom algorithms or software that are central to the research but not yet described in published literature, software must be made available to editors and reviewers. We strongly encourage code deposition in a community repository (e.g. GitHub). See the Nature Research guidelines for submitting code & software for further information.

Data

Policy information about availability of data

All manuscripts must include a data availability statement. This statement should provide the following information, where applicable:

- Accession codes, unique identifiers, or web links for publicly available datasets
- A list of figures that have associated raw data
- A description of any restrictions on data availability

Microarray data generated in this study are deposited at NCBI GEO (https://www.ncbi.nlm.nih.gov/geo/query/acc.cgi?acc=GSE156186). Public domain data used in this study are from HUGO Gene Nomenclature Committee (https://www.genenames.org), HomogoGene (https://www.ncbi.nlm.nih.gov/homologene), and SFARI Gene (http://www.sfari.org).

Field-specific reporting

Please select the one below that is the best fit for your research. If you are not sure, read the appropriate sections before making your selection.

- [x] Life sciences
- [ ] Behavioural & social sciences
- [ ] Ecological, evolutionary & environmental sciences

For a reference copy of the document with all sections, see nature.com/documents/nr-reporting-summary-flat.pdf

Life sciences study design

All studies must disclose on these points even when the disclosure is negative.

Sample size	No statistical methods were used to pre-determine sample sizes, but the sample sizes were similar to those in previous publications (spine density analysis: Sasaki et al. Brain Struct. Funct. 220, 3245 [2015]; patch clamp recording: Antoine et al. Neuron 101, 648 [2019]; LTD: Sawtell et al. J. Neurophysiol. 82, 3594 [1999]; spine volume analysis: Yasumatsu et al. J. Neurosci. 28, 13592 [2008]; vocalization: Takahashi et al. Science 349, 734 [2018]; transcriptome: Zhang et al. Reprod. Toxicol. 77, 53 [2018]).
Data exclusions	Patch clamp data with a series resistance > 28 MΩ (0M) or >18 MΩ (3M and 6M) or those with an unstable baseline were excluded. Field EPSP data were excluded if the baseline EPSP amplitude was unstable. Microarray samples with a MAS5 absent call > 0.4 were excluded.
Replication	All experiments were replicated multiple times, and similar results were reproduced. Numbers of replication are in the manuscript.
Randomization	Pregnant marmosets were randomly assigned for valproic acid administration. The offspring were selected for experiments without knowledge about health conditions or behavioral traits.
Blinding	The behavioral recording was not performed blind to the experimental conditions because it does not involve intervention by the investigator. The behavioral data were analyzed by a blinded investigator. Acquisition and analysis of electrophysiological and structural data were not performed blind to the experimental conditions. However, the electrophysiological data were analyzed using an automated algorithm that did not require intervention by the investigator. Also, the core results of the structural analysis were confirmed by multiple investigators.

Reporting for specific materials, systems and methods

We require information from authors about some types of materials, experimental systems and methods used in many studies. Here, indicate whether each material, system or method listed is relevant to your study. If you are not sure if a list item applies to your research, read the appropriate section before selecting a response.

Materials & experimental systems

n/a	Involved in the study	
x		Antibodies
x		Eukaryotic cell lines
x		Palaeontology and archaeology
	x	Animals and other organisms
x		Human research participants
x		Clinical data
x		Dual use research of concern

Methods

n/a	Involved in the study	
x		ChIP-seq
x		Flow cytometry
x		MRI-based neuroimaging

Animals and other organisms

Policy information about studies involving animals; ARRIVE guidelines recommended for reporting animal research

Laboratory animals	Marmoset (Callithrix jacchus) of both sexes, 0, 3, and 6 months old.
Wild animals	No wild animals were used.
Field-collected samples	No field-collected samples were used.
Ethics oversight	All experiments were approved by the Animal Research Committee of the National Center of Neurology and Psychiatry and the Animal Care and Use Committee of the National Institute of Radiological Sciences, and were in accordance with the NIH Guide for the Care and Use of Laboratory Animals.

Note that full information on the approval of the study protocol must also be provided in the manuscript.
